# The Influence of Gender Equality on Volunteering Among European Senior Citizens

**DOI:** 10.1007/s11266-021-00443-6

**Published:** 2022-01-04

**Authors:** Julia Sánchez-García, Ana Isabel Gil-Lacruz, Marta Gil-Lacruz

**Affiliations:** 1 Department of Business Direction and Organization, University of Zaragoza, Zaragoza, Spain; 2 Department of Psychology and Sociology, University of Zaragoza, Zaragoza, Spain

**Keywords:** Volunteering, Equality, Welfare system, Age, Organisations

## Abstract

This research analyzes how gender equality influences the participation of European senior citizens in a range of volunteering activities (Social Awareness, Professional and Political, Education, and Religion). The main contribution is the simultaneous consideration of different levels of data aggregation: individual, national and welfare system. This allows conclusions to be drawn on the effects of variables linked to sociodemographic characteristics, gender equality and welfare systems. The empirical estimation utilised microdata from the World Values Survey (2005/09 and 2010/14) and the United Nations Development Programme. Results suggest that the European senior citizens appear to believe that they are more equal than the official statistics of their countries indicate. Men are more likely to participate in professional and education activities; women are more likely to be involved in religious organisations. Welfare systems influence volunteering behaviours. The promotion of macro-policies for gender equality could be important for increasing participation in non-profit organisations.

## Introduction

The "active aging" framework has become one of the main policy responses to demographic challenges in older age (World Health Organization, 2012). Active aging could be promoted by social participation in community through volunteering. Volunteering in elderly people has positive effects (Gil-Lacruz et al., 2018; Krause & Rainville, 2018). Among its benefits, research has revealed that senior volunteers are more protected from health risks related to retirement and physical inactivity than those who do not volunteer (Gil-Lacruz et al., 2019; Wilson & Musick, 2012). According to data of Volunteurope report (2012), the percentage of elderly people in volunteering varies substantially among the European countries, with high rates in the Netherlands (20%), and low rates in Greece (3%). Specifically, Boccacin and Lombi (2018) found that older women volunteer more in Poland, Ireland, and Spain, while older men in Belgium, France, and Germany.

Gender differences in civic engagement are often analyzed as a social construct (Boccacin & Lombi, 2018). The conceptual approach frequently used is the theorical framework of social learning (Bandura, 1969). The explanation is that people reproduce and imitate observed gender role models. Although the literature reviewed shows that women are more involved in volunteering than men (Mesch et al., 2006; Wymer, 2011), this result loses intensity when socio-economic factors are controlled, such as income level and family composition (Hook, 2004; Taniguchi, 2006). These differences suggest that certain individuals and macrosocial factors may exert a marked influence on senior volunteering between genders. Nevertheless, as noted by Gil-Lacruz et al. (2019), studies conducted in countries have been focused on the positive effects related to volunteering (e.g., well-being, health, and satisfaction), ignoring others individual and macrosocial factors that account for civic engagement. Consequently, in the current research, the main objective is to analyze the individual and contextual determinants of senior volunteering, especially those that are gender-related, and on their interaction with macrosocial-level predictors.

The main contributions of the research are: (1) a gender perspective in the study of senior volunteering that considers sociocultural, sociodemographic and socioeconomic factors; (2) the analysis of influence of gender equality through two factors: the *Attitude Towards Gender Equality* (subjective ideological indicator) and the *Gender Equality Index* (objective structural indicator); and, (3) the European comparative study of how welfare system models (Continental, Nordic, Mediterranean and Eastern) influence four categories of volunteering: Social Consciousness, Professional and Political, Education and Leisure, and Religion. Data was taken from the World Values Survey (WVS; 2005/09, and 2010/14) and the United Nations Development Program (UNDP, 2005/14). The empirical estimation is carried out considering simultaneously different levels of data aggregation: Individual, National and Welfare Systems.

## Literature Review

### Volunteering

Volunteering is defined as any unpaid activity in which "…time is given freely to benefit another person, group or organisation" (Wilson, 2000, p. 215). The scientific literature provides distinct explanations for this behaviour (Musick & Wilson, 2008; Studer & von Schnurbein, 2013) but there are no clear conclusions regarding a specific volunteer profile (Norris & Inglehart, 2006). In addition to the heterogeneity of the population studied, the terminology [volunteer, volunteering, volunteerism] include a wide range of activities that are carried out by numerous organizations with different causes. Wilson and Musick (2012) show three main areas of research on volunteering participation as “the antecedents” …” experiences” …and “consequences”.

In this research, we analyze the antecedents of volunteering, which is referred to what influences an individual to select one type of organisation to volunteer instead of another type of organisation. We only examinate the effect of formal volunteering in different organizations in our study because it defines direct activities and can more accurately be measured.

### Individual Factors and Volunteering

Nichols and Shepard (2006) have shown that the relationship between voluntary participation and age corresponds to an inverted U: volunteering peaks in middle age and then begins to decline, whereas Wymer (2011) notes that volunteers are often older. According to life course theories, in middle age, volunteering has to do with family and work roles such as stable jobs and parenting that increase interest in the community (Flanagan & Levine, 2010). The values of predisposition to change can influence older adults to volunteer (Ariza-Montes et al., 2017). Motivations and personality traits, such as extraversion, appear to be relevant in deciding to volunteer (Kwok et al., 2013). Other factors such as the sense of belonging to the community are decisive in being a volunteer among the elderly (Pozzi et al., 2014). However, the factors that influence how seniors choose to volunteer are not conclusive, more research is needed to strengthen their participation (Boccacin & Lombi, 2018; Gil-Lacruz et al., 2019).

The study on whether men or women are more likely to volunteer has yielded contradictory results (Downward et al., 2020; Taniguchi, 2006; Wilson & Musick, 2012). Data from Bureau of Labor Statistics (2009) suggest more women are involved in activities than men in US, while in Sweden, there are more male volunteers than female (Musick & Wilson, 2008). These results vary depending on the type of activity. Some categories have strong gender norms, while in other areas gender differences would not be relevant (Fyall & Gazley, 2015). A recent European study has found a positive relationship in the decision to volunteer in awareness and social justice activities for women, while in professional activities for men (Gil-Lacruz et al., 2018). This may be due to social expectations about what activities are appropriate for women and men (see West & Zimmerman, 1987, for a further review of "doing gender").

Gender differences in volunteering could be explained by gender roles (Eagly, 1987). Due to the socially expected role of men as breadwinners, unemployment inhibits men's volunteering as they devote their time to seeking paid employment to fulfil their role as primary breadwinner (Taniguchi, 2006). In contrast, unemployed women may find participation in volunteer organizations more acceptable, as long as their family members are well cared for (Taniguchi, 2006). However, women devote more hours to household chores than men and provide more informal support (Hook, 2004), so these additional responsibilities may hinder women's access to volunteering and their time to volunteer. Albeit that the above results focus on middle-aged people, it is unknown whether this pattern is perpetuated among older people.

Given that socialization in many countries has changed over time in progress toward equality and occurs at a young age, Wemlinger and Berlan (2016) report that there are age differences between women and men who volunteer. On the one hand, gender equality has a great impact on young people who feel less constrained by traditional gender roles than precedent generations. On the other hand, progress in equality has less impact on seniors as they have experienced a greater degree of gender socialization than the youngest.

Previous literature has highlighted that socio-economic status and educational level are positively correlated with volunteering (Gil-Lacruz, et al., 2019). In addition to individual factors, other contextual factors could be influencing how gender differences are reflected in senior volunteering (Fyall & Gazley, 2015; Gil-Lacruz et al., 2018). We are interested in considering how social factors related to gender equality and welfare systems influence volunteering by older people.

### Macrosocial Factors and Volunteering

Despite the marked tendency to focus on consequences of volunteering in elderly, several theorists have considered macrosocial factors that could be key to understanding senior volunteering. For instance, some authors have recently shown that contextual factors, such as the government expenditures on health may reduce volunteer rates, whereas government expenditures on other social issues could reinforce them (Gil-Lacruz et al., 2018, 2019). Veal and Nichols (2017) showed that higher levels of volunteering in European countries are associated with lower levels of income inequality. Researchers have reported that other macro-social factors, such as the COVID-19 pandemic, could be positively related to senior volunteering (Chan et al., 2021). However, the results for test of other structural factors in relation to age and gender are less convincing (Phillips, 2015).

Feminist scholars (Rotolo & Wilson, 2007; Wemlinger & Berlan, 2016) have maintained that the main cause cause of the gender gap in employment are due to macro-social norms related to gender and the resulting organisational segregation (Fontanella, et al., 2020). Few studies have empirically examined the relation between gender-related macrosocial indicators and volunteering (Fyall & Gazley, 2015; Wellinger & Berlan, 2016). Following the *volunteering process model* (Snyder & Omoto, 1992; Wilson & Musick, 2012), in relation to antecedents, we focused on the social context through two kinds of factors: (a) one ideological factor belonging to the macro system related to norms, beliefs, and values about the gender roles in society; and (b) two structural indicators belonging to be macro system related to gender equality and countries. Specifically, we examined one gender-related ideological macrosocial factor (attitudes toward gender equality) and two structural macrosocial factors (indicator of gender equality and welfare systems).

In this sense, the United Nations (2018) points out the importance of the concept of gender equality referred to equal rights and opportunities (in work, education, access to resources, etc.) to achieve sustainable development. Despite the apparent relevance of attitudes toward gender equality in work highlighted by international organizations, such as the United Nations (2018), we have not found many studies that examine their role in volunteering and from a cross-cultural point of view. Wemlinger and Berlan (2016) have found that less acceptance of traditional gender roles facilitates and promotes women’s advancement in volunteering. For instance, increasing women's volunteerism in organisations commonly associated with men such as sports and politics (Boje et al., 2019). Therefore, attitudes which reject gender roles could be relevant in the position of men and women in society and in non-profit organizations (Shantz et al., 2019).

In the current study, we analyzed the relation between volunteering in different organizations and a structural gender-related macrosocial factor using Gender Inequality Index [GII]. The GII indicator “measured the gender gaps in three aspects of human development—reproductive health, empowerment and the labor market—and exposes differences between men and women in the distribution of achievements” on a scale from 0 (full equality) to 1 (total inequality)” (United Nations Development Program, 2005/14). There is empirical evidence for the relation between structural indicators of gender equality and volunteering (Fyall & Gazley, 2015). For instance, the occupational status of women in a given country has been linked to volunteering, specifically, in professional and social justice activities (Gil-Lacruz & Saz-Gil, 2018).

Previous literature has explored a wide variety of individual characteristics to explain cross-country differences in volunteering ratios (Gil-Lacruz et al., 2018, 2019; Wilson, 2000). Meanwhile a high female participation at age 55 + is found in Latvia, Greece, Poland and Spain, a high male participation at age 55 + is found in Belgium, the Netherlands, France and Germany (Boccacin & Lombi, 2018). In relation to activities, in countries with gender inequality, there is segregation in the type of organizations in which men and women participate (Wemlinger & Berlan, 2016), with men volunteering more in instrumental organizations, while women volunteer more in expressive organizations. Plagnol and Huppert (2009) concluded that differences in volunteering ratios between countries could not be fully explained by differences in individual social, psychological or cultural factors associated with volunteering. Macrostructural explanations for cross-country variations in volunteering are therefore necessary to understand national patterns of individual behaviour.

In this sense, Salamon and Anheier's (1998) theory of social origins points out that welfare systems could represent a relevant factor in volunteering. Different institutional regimes are characterized by different patterns of redistribution and social security, qualities of civil liberties, pluralism and rule of law (Enjolras, 2021). Therefore, welfare systems cannot be reduced only to the product of the unilateral extension of a single factor, such as income inequality, size of the non-profit sector, diversity, education or industrialization (Esping-Andersen, 1990), but they represent broad social structures comprising complex interrelationships between social institutions and cultural, social and economic factors.

In relation, a data analysis conducted in the Volonteurope report in 2012 compared senior volunteering ratios among European countries. Meanwhile in Sweden, with a strong welfare system, elderly are more likely to volunteer in expressive activities (culture and advocacy groups), in the UK, with a government with a weaker role in the provision of welfare-related services, the participation of the elderly in care associations is more prevalent. Consequently, each welfare system generates different sets of capabilities that, in turn, encourage or inhibit the participation in volunteer activities (Enjolras, 2021).

### The Current Study

We explore the role of individual and macrosocial factors related to gender and welfare systems in senior volunteering. Specifically, we had three objectives: (a) to evaluate whether the ideological and structural factors related to gender inequality are significantly associated with senior volunteering, (b) to explore the extent to which these macrosocial factors interact with gender in predicting volunteering and (c) to analyze whether welfare systems influence volunteering. Given only a few researchers have analyzed the impact of these variables on different types of volunteering, we conducted separate tests of models for general volunteering, social awareness, professional and political, education and leisure, and religion volunteering.

We used two data sources: (1) a database from World Values Survey (WVS, waves 2005/09 and 2010/14), which was used to obtain the dependent variables (volunteering and categories), the ideological variable (attitudes toward gender equality), and individual predictors (age, gender, income level and education level); (2) the Gender Inequality Index (GII; United Nations Development Program; 2005/14), which we used to obtain data at the country level.

We hypothesized (Hypothesis 1) that the selected individual variables would be significantly related to volunteering: Income level and education level would have a positive influence on volunteering. Moreover, we hypothesized (Hypothesis 2) that at the macro level, the frequency of volunteering would be higher in countries with more attitudes toward gender equality (Hypothesis 2a), in countries with lower GII (Hypothesis 2b) and in countries with a Nordic welfare system (Hypothesis 2c).

We explored whether older women volunteer more than men in general and whether there are differences between organisations. Given that the evidence on age and gender is mixed, we decided not to form specific hypotheses on this. Also, we explored whether women would volunteer more in countries with higher attitudes towards equality and GII. In relation, we explored the differences between the two measures of equality between welfare systems. Finally, because research examining multilevel models specific to different types of volunteering is not extensive, no clear evidence exists on which to form separate hypothesis for each type of volunteering (social awareness, professional and political, education and leisure, and religion), and we considered these aspects of the study exploratory.

### Data

The empirical estimation utilised data from the World Values Survey (WVS); and from the United Nations Development Program (UNDP). The WVS is a cross-national survey conducted since 1990. The fourth and fifth waves have been utilized due to the availability of the variables referring to volunteering behaviour, attitudes towards gender equality and sociodemographic variables. It is a robust instrument for the analysis of volunteering. The study concentrated on European countries as they are homogeneous enough to make inferences from the results. The macrodata on Human Development from UNDP provided a gender equality index for the countries studied in the time period.

The sample comprised 8234 people aged 50–80, from 8 countries (Cyprus, Germany, the Netherlands, Poland, Romania, Slovenia, Spain and Sweden), for two-time waves (2005–2009 and 2010–2014).

The WVS looks at participation in 8 voluntary organisations. This paper analyzes volunteering at two aggregation levels: volunteering and type or category of activity. About type of categories of volunteering, we selected Sardinha (2011) model: (1) Social Awareness (environmental, humanitarian or charitable organisations); (2) Professional and Political (professional organisations, political parties and trade unions); (3) Education and Leisure (sports and recreation clubs, arts, music and educational organisations); (4) Religion (religious organisations or churches).

The explanatory demographic variables were gender and age^1^ (between 50–64 and 65–80); the socioeconomic variables included educational level and income.

Two explanatory variables analyzed gender equality: one is subjective ideological and the other is objective structural. As subjective ideological variable, attitudes towards gender equality were obtained through items from the WVS (*Men make better political leaders than women do; University is more important for a boy than for a girl; Men make better business executives than women do; Being a housewife just as fulfilling*). As objective structural variable, the Gender Inequality Index (GII) was obtained from the United Nations Development Program (0–1). The higher the value, the greater is the gender inequality. Since the subjective measure was an equality index, the objective index was made positive, in order to facilitate the interpretation of the results (*ObjectiveGenderEquality* = 1 − *ObjectiveGenderInequality*).

Countries were grouped by welfare systems (Gil-Lacruz & Marcuello, 2013; Sardinha, 2011): (1) Nordic—Sweden; (2) Continental—Germany, the Netherlands; (3) Mediterranean—Cyprus, Spain; (4) East—Poland, Romania and Slovenia. There were 8 geographical dummy variables for each country and 4 for each welfare system. There were also 2 dummy variables for each wave (2005–2009 and 2010–2014). The set of dummy variables allows the calculation of the geographic and temporal effects.

### Empirical Framework

Due to the binary nature of the volunteer dependent variables (1 active; 0 inactive) and the hierarchical organisation of data levels (individuals, national and welfare systems), the empirical study was based on multilevel Logit models (STATA: xtmelogit). Multilevel regression models serve a single dependent variable at the lowest level of disaggregation (individual) and incorporate a set of explanatory variables for each level (micro and macro). The study focused on volunteering as a dependent variable, and the micro variables (age, sex, educational level, and income) and macro variables (attitudes toward gender equality, GII and welfare system) which might explain volunteering. The results measured the sensitivity of the dependent variable when there is change in an explanatory variable. The parameters shown in the tables indicate the statistical significance, the sense and intensity of the effects of the independent variables.

The estimation analysis takes into account a non-linear response model; the data were structured for 8234 people ($i = 1, \ldots , 8 234$) corresponding to 8 European countries ($j = 1, \ldots , 8$). The probability of volunteering (${\text{Unpaidwork}}_{ij}$) was estimated as

1 $${\text{Unpaidwork}}_{ij} = X_{ij}^{^{\prime}} \beta_{j} + u_{j} + e_{ij}$$

where $X$ includes $K$ regressors ($K - 1$ variables and a constant) and the term error is characterized by $e_{ij} \approx N\left( {0, \sigma^{2} } \right)$. The model incorporates as many fixed effects ($\beta$) as there are random effects ($u$). The fixed effects indicate how the explanatory variables affect the decision to volunteer; the random effects include the variability between countries.

The estimates were repeated for each of the four categories ($f = 1$ social awareness; $f = 2$ professional and political; $f = 3$ education and leisure; $f = 4$ religion). The estimates were made independently: the same person can participate in different volunteering activities, so the categories are not exclusive:

2 $${\text{Unpaidwork}}_{fij} = X_{ij}^{^{\prime}} \beta_{fj} + u_{fj} + e_{fij}$$

The objective was to achieve the most accurate estimate of *β*. Estimates used 3 models: Model 1 included sociodemographic data (age, gender, educational level, and income) and temporal variables (2005–2014); Model 2 incorporated information on gender equality with a subjective variable (*Attitude towards gender equality*) and an objective variable (*GII*). The incorporation of the subjective and objective allows an examination of the egalitarian attitude of a population and the country's objective level of gender equality, something that could be decisive in explaining a greater or lesser occupational segregation in volunteering decisions. As explanatory determinants, two further interactions were considered: gender and subjective and objective equality measures. The interactions between the variables allow conclusions to be drawn on the relationship between the gender equality measures and the volunteering decisions of men and women. Model 3 added information on welfare systems (Nordic, Continental, Mediterranean and Eastern). The fact that the estimates were stable (in estimated value and significance) was a guarantee of robustness. Model 3 was justified with the results obtained by the ANOVA, which analyzes the dispersion of random effects according to groups of countries classified by welfare systems. The dispersion of random effects was greater between groups than intra-group, so the welfare system represents an adequate country classification model.

Estimates were carried out for men and women to consider differences between two subsamples. If the *β* estimation of an explanatory variable was different (in sense and/or intensity), there was evidence of differential gender effects.

## Results

Although the percentage of women (53%) was slightly higher than men (47%), the volunteer ratios were slightly higher for men (36%) than for women (33%). Professional and political volunteering was also higher for men than for women and volunteering with religious organisations was higher among women than men. There were no other relevant gender differences with regards to the other volunteering categories.

Women had lower scores at the secondary (33%) and tertiary (11%) educational levels compared to men (37% and 16%, respectively). As shown in Table 1, in the low-income group, the percentage of women is higher than that of men.

**Table 1:** First descriptive analysis. *Source* World Values Survey and UNDP

	Total		Female		Male	
	Mean (%)	SD	Mean (%)	SD	Mean (%)	SD
*Dependent variables*						
UnpaidWork	34.4	0.48	32.9	0.47	36.1	0.48
UnpaidSocialAwareness	6.3	0.24	6.4	0.24	6.3	0.24
UnpaidProffessionalPolitical	9.8	0.30	7.0	0.26	12.8	0.33
UnpaidHumanitarianCapital	18.8	0.39	17.0	0.38	20.8	0.41
UnpaidReligion	11.8	0.32	13.6	0.34	9.9	0.30
*Independent variables*						
Female	52.8	0.50	100.0	0.00	0.0	0.00
Male	47.2	0.50	0.0	0.00	100.0	0.00
Age_50–64	58.9	0.49	59.0	0.49	58.8	0.49
Age_65–80	41.1	0.49	41.0	0.49	41.2	0.49
Primary&NoStudies	40.8	0.49	42.2	0.49	39.3	0.49
SecondaryStudies	35.1	0.48	33.3	0.47	37.1	0.48
TertiaryStudies	13.7	0.34	11.5	0.32	16.1	0.37
LowIncome	31.3	0.46	35.7	0.48	26.6	0.44
MiddIeIncome	59.4	0.49	56.6	0.50	62.4	0.48
HighIncome	9.3	0.29	7.7	0.27	11.0	0.31
SubjectiveGenderEquality	70.2	0.25	72.1	0.24	68.0	0.25
ObjectiveGenderEquality	85.6	0.10	85.2	0.10	86.1	0.10
Nordic	10.5	0.31	10.3	0.30	10.8	0.31
Continental	37.0	0.48	35.3	0.48	38.9	0.49
Mediterranean	16.8	0.37	16.5	0.37	17.2	0.38
East	35.7	0.48	38.0	0.49	33.2	0.47
Wave_2005–2009	46.1	0.50	45.8	0.50	46.6	0.50
Wave_2010–2014	53.9	0.50	54.2	0.50	53.4	0.50

Countries have been aggregated by welfare systems: Nordic countries (Sweden), Continental countries (Germany and Netherlands), Mediterranean countries (Cyprus and Spain) and Eastern countries (Poland, Romania and Slovenia)

Table 2 shows the probability estimates of volunteering, taking into account the different waves and welfare systems for objective and subjective measures based on gender equality. Residents of the Nordic countries have the highest percentages on both measures, in contrast to the Eastern countries. The results show a positive progression over the years in greater subjective egalitarian attitudes in the population. Similarly, the objective measure of gender equality has also increased. Nevertheless, the percentages of subjective measure are higher than the percentages of objective changes occurring in societies progressing toward equality (e.g., reproductive health, labor force, and empowerment). The gap between the two indicators was greater for less egalitarian countries. For example, for the second wave, the differences among both indexes were higher in Mediterranean countries (20%) than in Nordic countries (9%).

**Table 2:** Descriptive analysis for welfare systems, gender equality and waves. *Source* World Values Survey and UNDP

	Objective gender equality (%)	Subjective gender equality (%)	Difference (%)
*Nordic*			
2005–2009	83.9	94.8	10.9
2010–2014	86.1	95.2	9.1
*Continental*			
2005–2009	72.7	89.2	16.5
2010–2014	76.3	92.9	16.6
*Mediterranean*			
2005–2009	68.7	87.3	18.6
2010–2014	68.7	88.6	19.9
*East*			
2005–2009	58.9	75.2	16.3
2010–2014	61.8	76.5	14.6

Countries have been aggregated by welfare systems: Nordic countries (Sweden), Continental countries (Germany and Netherlands), Mediterranean countries (Cyprus and Spain) and East countries (Poland, Romania and Slovenia)

Table 3 shows the aggregated estimates of the probability of volunteering. Table 4 shows the estimates according to the volunteering categories.

**Table 3:** Estimations for *UnpaidWork*: Xtmelogit

	Model 1	Model 2	Model 3
*Fixed effect*			
Female	− 0.018	− 1.396***	− 1.399***
Male^a^	–	–	–
Age_50–64	0.077	0.070	0.072
Age_65–80^a^	–	–	–
Primary&NoStudies^a^	–	–	–
SecondaryStudies	0.332***	0.340***	0.343***
TertiaryStudies	0.817***	0.813***	0.806***
LowIncome^a^	–	–	–
MiddIeIncome	0.254***	0.247***	0.248***
HighIncome	0.455***	0.453***	0.458***
SubjectiveGenderEquality	–	− 0.039	− 0.044
Female*SubjectiveGenderEquality	–	− 0.155	− 0.154
ObjectiveGenderEquality	–	4.098***	2.901***
Female*ObjectiveGenderEquality	–	1.729***	1.733***
Nordic^a^	–	–	–
Continental	–	–	0.180
Mediterranean	–	–	− 0.501**
East	–	–	− 0.412
Wave_2005–2009	0.063	0.142***	0.123**
Wave_2010–2014^a^	–	–	–
Intercept	− 1.208***	− 4.729***	− 3.458***
*Random effects*			
*σ*^2^	0.560	0.284	0.150
LR test (Prob > chi^2^)	0.000	0.000	0.000
*Analysis of variance*			
Betweengroups	2 390.093	411.569	
Withingroups	842.337	258.119	
Bartlett’s test	0.000	0.000	

***, ** and * explanatory variables are statistically significant at 99, 95 and 90 % levels

^a^Variable of reference

**Table 4:** Estimations for *UnpaidWork by categories*: Xtmelogit

	SocialAwareness			Religion		
	Model 1	Model 2	Model 3	Model 1	Model 2	Model 3
*Fixed effect*						
Female	0.112	− 2.266*	− 2.261*	0.399***	− 1.167*	− 1.186*
Male^a^	–	–	–	–	–	–
Age_50–64	0.006	− 0.009	− 0.017	− 0.362***	− 0.360***	− 0.359***
Age_65–80^a^	–	–	–	–	–	–
Primary&NoStudies^a^	–	–	–	–	–	–
SecondaryStudies	0.732***	0.672***	0.691***	− 0.182**	− 0.067	− 0.074
TertiaryStudies	1.089***	1.022***	1.038***	0.020	0.199*	0.202*
LowIncome^a^	–	–	–	–	–	–
MiddIeIncome	0.299**	0.323**	0.332***	0.019	0.021	0.015
HighIncome	0.460***	0.474***	0.463***	0.055	0.064	0.076
SubjectiveGenderEquality	–	− 0.031	− 0.018	–	− 1.034***	− 1.019***
Female*SubjectiveGenderEquality	–	0.440	0.457	–	− 0.208	− 0.209
ObjectiveGenderEquality	–	4.327***	6.052***	–	0.131	0.352
Female*ObjectiveGenderEquality	–	2.304*	2.284*	–	2.061**	2.086**
Nordic^a^	–	–	–	–	–	–
Continental	–	–	− 0.411**			0.833***
Mediterranean	–	–	0.089			0.370**
East	–	–	0.192			0.463**
*Random effects*						
*σ*^2^	0.601	0.263	0.117	0.249	0.243	0.000
LR test (Prob > chi^2^)	0.000	0.000	0.124	0.000	0.000	1.000
*Analysis of variance*						
Betweengroups	1 382.027	202.330		498.651	474.733	
Withingroups	1 975.076	255.582		34.933	11.898	
Bartlett’s test	0.000	0.000		0.000	0.000	

	Professional & political			Education & leisure		
	Model 1	Model 2	Model 3	Model 1	Model 2	Model 3
*Fixed effect*						
Female	− 0.578***	− 1.599**	− 1.578**	− 0.113*	− 4.657***	− 4.676***
Male^a^	–	–	–	–	–	–
Age_50–64	0.649***	0.593***	0.593***	0.138**	0.085	0.086
Age_65–80^a^	–	–	–	–	–	–
Primary&NoStudies^a^	–	–	–	–	–	–
SecondaryStudies	0.434***	0.419***	0.428***	0.460***	0.395***	0.401***
TertiaryStudies	1.171***	1.108***	1.108***	0.896***	0.794***	0.790***
LowIncome^a^	–	–	–	–	–	–
MiddIeIncome	0.385***	0.342***	0.339***	0.425***	0.407***	0.410***
HighIncome	0.381***	0.371**	0.363**	0.599***	0.580***	0.588***
SubjectiveGenderEquality	–	− 0.018	− 0.029	–	0.466**	0.467**
Female*SubjectiveGenderEquality	–	0.684*	0.682*	–	0.581*	0.583*
ObjectiveGenderEquality	–	1.008	1.045	–	7.321***	6.015***
Female*ObjectiveGenderEquality	–	0.601	0.578	–	4.491***	4.510***
Nordic^a^	–	–	–	–	–	–
Continental	–	–	− 0.477	–	–	0.687**
Mediterranean	–	–	− 0.237	–	–	− 0.544*
East	–	–	− 0.303	–	–	− 0.397
*Random effects*						
*σ*^2^	0.339	0.281	0.231	1.099	0.522	0.236
LR test (Prob > chi^2^)	0.000	0.000	0.001	0.000	0.000	0.000
*Analysis of Variance:*						
Betweengroups	284.206	139.490		9780.603	1773.972	
Withingroups	513.178	321.567		2889.483	555.705	
Bartlett’s test	0.000	0.000		0.000	0.000	

Estimations have also been controlled by age, welfare systems and waves. These results have been omitted to improve presentation, but they are available on request

***, ** and * explanatory variables are statistically significant at 99, 95 and 90 % levels

^a^Variable of reference

In Model 1, the degree of variance of the random effect, *σ*^2^_*u*,_ is significant; this justifies the analysis of the dependent variable with a Multilevel Logit model, in that the decision to volunteer is explained by fixed and random effects. Demographic and socioeconomic variables are included in the fixed effects. Volunteering decisions correlate positively with secondary and tertiary education levels and a medium and high income (Hypothesis 1). For random effects, the ANOVA estimate identified the differences between countries and welfare systems. The variance of the random effects was greater among countries with different welfare systems than among countries with similar welfare system. This result supports the use of welfare systems as a country classification method.

The subjective ideological and objective structural variables related to gender equality are introduced in model 2 (Hypothesis 2a and b). The coefficients of the individual variables considered in Model 1 remain stable as a sign of the robustness of the results. The GII positively predicted volunteering while no effect of attitudes towards equality was found. Women report fewer volunteering activities compared to men. It is clear that the objective gender equality measure positively influences the volunteer ratios, particularly for women. With random effects, variance for the differences between countries is significantly reduced with respect to Model 1, especially for countries with different welfare systems. It can be concluded that the introduction of the objective macro variable as a fixed effect reduces the unexplained variability between countries. The results obtained by ANOVA for Models 1 and 2 justify the introduction of welfare systems as an explanatory variable of the decision to volunteer.

Welfare systems are included in Model 3 (Hypothesis 2c). The estimated coefficients are similar to the two previous models, so the results are robust. Living in a Mediterranean country has a negative impact on volunteering ratios when compared to living in a Nordic country. This result is important because geographical divergence persists when controlled by a series of explanatory variables.

Table 4 replicates the same procedure for the categories of volunteering. In Model 1 it can be seen that women between 65 and 80 are more likely to participate religious volunteer organisations; men between 50 and 64 are more involved in professional and political groups and educational and leisure activities. All categories of volunteering were positively related to secondary and tertiary education level, and medium and high incomes, with the exception of religion. In terms of random effects, variance was greater between countries with different welfare systems with regards to religion, education and leisure. Variance in countries with the same welfare system was greater for professional and political activities and social awareness.

Model 2 introduced subjective ideological and objective structural variables related to gender equality. The coefficients of the individual variables contemplated in Model 1 remained stable, except for gender in volunteering in areas of social awareness and religion. The attitudes toward gender equality measure positively influenced the ratios of volunteering for education and leisure and had a negative effect on religion. The GII measure was positively related to volunteering ratios for social awareness, education and religion activities.

The GII shows a positive correlation between women and social awareness, education and religion volunteering. The introduction of attitudes toward gender equality negatively influences in religious volunteering although it seems to increase the participation of men in this activity. Women who have a more positive attitude toward gender equality are more likely to volunteer in professional and political groups and in educational and leisure activities. Variance for the differences between countries is notably reduced with respect to Model 1, especially for social awareness, education and free-time activities. Consequently, the inclusion of the country-level explanatory variables related to equality has largely reduced the unexplained variability across countries.

Finally, Model 3 incorporated the welfare systems; the results were similar to the two previous models. In addition, welfare systems positively influenced rates of volunteering in religious organisations, especially in Continental countries, compared to Nordic countries. In Continental countries, there were lower rates of volunteering in social awareness activities, although rates were higher in activities related to education and free-time. In Mediterranean countries, there were fewer volunteers in the education and free-time categories compared to Nordic countries.

## Discussion

The results of this study provide new empirical evidence on international analysis of gender and senior citizen volunteering: (1) differences in volunteering between older men and older women were examined with integrated data at 3 levels: individual, national, and welfare systems. (2) gender equality was considered at ideological and structural macrosocial level as an explanatory factor for the volunteering decisions of both genders (Wemlinger & Berlan, 2016). (3) the countries analyzed were grouped by welfare systems; this is relevant because volunteering depends on both individual and contextual factors (Gil-Lacruz & Marcuello, 2013). The findings of this research could be of interest for the achievement of the United Nations Sustainable Development Goals, specifically Goal 3*: Ensure healthy lives and promote well-being for all at all ages*. We understand that foresting volunteerism promotes active aging in pursuit of well-being.

Estimated coefficients relative to socioeconomic variables and the profile of the volunteer were similar to those found in previously published scientific literature (Gil-Lacruz, et al., 2018; Wemlinger & Berlan, 2016). Income and educational level correlated positively with volunteering as we expected. In the descriptive data, men were shown to be more involved with volunteering than women, which was statistically significant in the estimates of general volunteering and its different types. However, when we consider measures of equality, women participate to a greater extent. This shows that in countries with a good access and opportunities for education and employment for women, reduced household responsibilities and high levels of education promote female civic participation (Wemlimger & Berlan, 2016).

The data reveals that there are no significant differences between the two age groups in the general volunteering ratios. Nevertheless, the results show a higher participation in professional and political organizations, education and leisure among men, those with higher educational level and among people aged 50–64 years; while a higher participation in religious associations among women, those with lower educational level and among people aged 65–80 years. Therefore, the relationship between volunteering and age could depend on the type of voluntary activity (Nichols & Shepard, 2006).

Men aged 50–64 might be more likely to volunteer than men aged 65–80 because they are of active working age (Taniguchi, 2006), report higher health status (Gil-lacruz et al., 2019), greater use of technology and social networks, and higher financial security (Boccacin & Lombi, 2018). The latter is consistent with the results of Gil-Lacruz et al. (2018), in which income level is positively correlated with volunteering, especially professionally and educationally. In contrast, the fact that women between the ages of 65 and 80 volunteer only in religion might be due to factors such as being a housewife or retired, or devoting their efforts in informal care, which reduce the likelihood of volunteering in other categories (Boccacin & Lombi, 2018; Gil-Lacruz & Marcuello, 2013).

Since the 1990s, gender equality issues have been given greater consideration by recognizing women's social rights, improving their access to education and employment, and increasing their social participation (Costa, 2014). Social advancement is mostly experienced by younger age groups (Shantz et al., 2019; Wemlinger & Berlan, 2016). The older age groups we analyze have already experienced a strong degree of gender socialization (Lott, 1981). This allows us to explain why these improvements are not reflected in the older ones. In relation, Einolf (2011) found that gender differences in volunteering still exist among the youngest. Consequently, the importance of gender roles in any age group remains.

The attitude towards gender equality and GII are associated, as predicted, with higher ratios of volunteering. In more egalitarian contexts men are more likely to approach traditionally female organizations, while women are less likely to approach traditionally male organizations. This means that the advancement is more progressive for men in unpaid employment. The subjective measure is positively correlated for men in general volunteering, and for women in professional and political activities. Nevertheless, it is not supported by objective measurement. Therefore, the objective barriers faced by women in accessing instrumental organizations continue to exist (Fontanella et al., 2020). Inglehart and Norris (2003) explain that in countries with less objective gender equality, women's political activism is suppressed due to strong female role beliefs, which reduces the likelihood that they will volunteer in professional and political organizations. Consequently, attitudes in advancing equality among women is not enough. It is necessary to extend them to the entire population, as well as to achieve objective markers of progress.

Regarding welfare systems, the inhabitants of the Nordic countries are more voluntary than those of the Mediterranean countries, as expected. In line with Esping-Andersen (1990), the Nordic model is characterized by extensive universal social rights pursuing equality and very limited private welfare provision, which encourages people to be active. Moreover, the fact that Nordic citizens have more egalitarian attitudes and the country reports a high rate of gender equality in employment, health and empowerment has a positive impact on higher volunteering rates than Mediterranean countries. Specifically, high ratios of volunteering to social awareness and education are in line with Salomon and Anheier's (1998) theory that volunteer organizations are vehicles for expressing social or recreational interests.

However, we did not observe low volunteer ratios in the Continental and Eastern model as Salamon and Anheier (1998) did. It could be because they included a different set of countries. For instance, in the Eastern model they only considered Japan, while we included European countries such as Romania, Poland and Slovenia. Therefore, different cultures can lead to discrepant results within the same welfare system. Moreover, it could be due to their current historical and economic evolution, or the indicators used to measure their differences (e.g., public spending), something that has already been recognized later by the authors (Anheier et al., 2020).

Notwithstanding the above, we find that welfare systems are significant depending on the type of activity. For instance, the Continental model targets organizations related to education and leisure, while the Eastern model targets religious associations. Since each welfare system is dedicated to specific sectors, the regime theory can be complemented by others, such as the market failure/government failure theory (Weisbrod, 1977), which views voluntary participation as a response to the lack of public services to meet the demands of the population in certain sectors.

Finally, decisions about volunteering should be approached in a multidisciplinary framework, including social factors as relevant predictors of volunteering rates. The reason for including welfare systems is to advance the comparative study of the determinants of volunteering by carrying out a hierarchical model which integrates data at three levels of aggregation (individual, national and welfare system). Thanks to country and wave dummy variables we merge micro with contextual factors. Moreover, the fact that individual sociodemographic variables and national data reduce unobserved heterogeneity between and within welfare systems reveals the importance of their inclusion. The differences we have found in the volunteering ratios according to the gender of the elderly indicate that the real challenge for welfare systems seems to find the optimal combination of conciliation policies, according to national circumstances and having the desired organization in society, i.e., towards gender equality (Costa, 2014).

## Practical Implications

The practical implications of the paper show the need to improve the conditions and opportunities for social participation in older adults. As the research results show, different welfare systems in European national contexts can either facilitate or constrain the volunteering development of older people. For this reason, it is necessary to improve social inclusion in solidarity networks, increase the funds allocated to support voluntary activities in elderly, reduce the difficulty of traveling to a voluntary organization (ie, public transport) and improve the help that elderly offer to their family members, which in turn prevents them from engaging in formal volunteering.

## Limitations

The main limitation of the study is the analysis was cross-sectional, which only offers a static perspective. Research with panel data would allow in-depth content analysis, examining the changes that the subjects undergo over time. In addition, a work with panel data would explain unobserved heterogeneity. The absence of Anglo-Saxon countries in the analysis due to lack of available data, for example United Kingdom for both time waves, limits the generalition of the results to the European level. Besides, Sweden is the only country with available data to represent the Nordic welfare system.

Future research could look at the hierarchical positions held by women and men within voluntary organisations, with the aim of elucidating whether there is occupational segregation and how it can be influenced by the individual and national level of equality. The evidence from this study on different categories of volunteering and the gender gap could be complemented with information related to informal volunteering.

## Footnotes

1 In Europe, as in other economically advanced countries, there is a general tendency to delay the retirement age. 65 years is the reference point for this work as the database includes periods up to 2014. For future work, with subsequent databases, it might be necessary to reconsider the minimum age relative to retirement.
